# Evolution of Microbial Pathogens

**DOI:** 10.3201/eid1208.060579

**Published:** 2006-08

**Authors:** Jianping Xu

**Affiliations:** * McMaster University, Hamilton, Ontario, Canada

**Keywords:** evolution of microbial pathogens, book review, Jianping Xu

This book is one of the first to provide an up-to-date view on a fundamental issue in medical microbiology research: how the accumulated genetic and genomic information is contributing to our understanding of virulence factors and the evolution of virulence in microbial pathogens. The editors should be commended for assembling 35 outstanding contributors, who specialize in various areas of microbial pathogenesis and evolution.

The 14 chapters are grouped into 3 broad sections: general concepts in microbial evolution, environment and the evolution of microbial pathogens, and the evolution of selected pathogenic species and mechanisms. At the beginning of each section, a concise overview of individual chapters integrates the content of the chapters into the section.

In the first section, the 5 chapters introduce the basic processes affecting microbial evolution, from the individual molecular level to the genomic, cellular, and population levels. Well-known concepts such as horizontal (lateral) gene transfer, the relationship between virulence and transmission, and pathogenicity islands are discussed extensively. Of special note are 2 chapters that are often missing in traditional medical microbiology books: 1 describes how long-term experimental evolutionary studies in the laboratory can contribute to our understanding of microbial pathogen evolution in the environment and clinics, and the other describes how gene inactivation and gene loss can be creative forces during the evolution of many microorganisms, especially obligate intracellular pathogens.

In the second section, the 5 chapters review how interactions between microbes and various natural biotic and abiotic factors can influence the origin and evolution of virulence in microbial pathogens. These factors are the physical, chemical, and biologic properties of the soil environment; the plant and animal environments; and to a lesser extent, the aquatic and atmospheric environments. Other highly topical issues are the evolutions of toxins, secretion systems, and antibiotic resistance.

In the third section, the 4 chapters extensively discuss the evolution of selected groups of microbial pathogens: group A Streptococcus and Staphylococcus aureus; enteric pathogens such as Escherichia coli, Salmonella enterica, and Yersinia spp.; Mycobacterium spp.; and fungal pathogens such as Candida albicans and Cryptococcus neoformans. The authors provide rich detail of molecular variation within and between populations of these species and describe how patterns of population genetic variation have contributed to our understanding of the evolution of virulence and virulence factors in these pathogens.

I have no major criticism of what is included in this book; rather, I note what is absent, which could have made the book more comprehensive. The first is an overall evolutionary framework of the distribution of microbial pathogens on the phylogenetic tree. Such a macro-evolutionary framework would showcase the nonrandom patterns of the distribution of human pathogens among major phylogenetic groups of microorganisms. Second, although base substitutions, insertions and deletions, homologous recombination, and lateral gene transfer are discussed throughout the book, a generalized quantitative review of the relative contributions of these processes during the evolution of certain groups of microbial pathogens (e.g., E. coli) would have been highly informative. These processes are fundamental to the evolution of all groups of organisms, and the analysis of the unparalleled datasets in microbial pathogens can teach us much about the evolution of other groups of organisms. Third, although many human pathogens are globally distributed, a substantial number show geographic specificity and endemism. Therefore, the spatial and temporal patterns of distribution of microbial pathogens within a species and at the species level across the globe are highly relevant to the evolution of microbial pathogens. Lastly, this book is highly biased toward bacterial pathogens. Only 1 chapter deals with nonbacterial (fungal) pathogens, and no chapter discusses viral or protozoan pathogens, which are responsible for some of our biggest public health threats, e.g., HIV, influenza A, and Plasmodium falciparum.

Nevertheless, this is a timely and much-needed book about the evolution of bacterial virulence and its pathogenesis. It will be a valuable resource for researchers in the field of microbial evolution and pathogenesis, senior undergraduate students, graduate students, faculty who teach medical microbiology and microbial evolution, clinical microbiologists, and infectious disease specialists.

